# Use of Cumulative Assessments in U.S. Schools and Colleges of Pharmacy

**DOI:** 10.3390/pharmacy3020027

**Published:** 2015-06-12

**Authors:** Deepti Vyas, Jenana Halilovic, Myo-Kyoung Kim, Marcus C. Ravnan, Edward L. Rogan, Suzanne M. Galal

**Affiliations:** University of the Pacific, Thomas J. Long School of Pharmacy and Health Sciences, 751 Brookside Road Stockton, CA 95207, USA; E-Mails: jmaker@pacific.edu (J.H.); mkim@pacific.edu (M.K.K.); mravnan@pacific.edu (M.C.R.); erogan@pacific.edu (E.L.R.); sgalal@pacific.edu (S.M.G.)

**Keywords:** comprehensive assessment, cumulative assessment, milestone examination, progress assessment

## Abstract

The Accreditation Council of Pharmacy Education (ACPE) has taken a strong stance on assessment in pharmacy education. One available assessment tool is cumulative assessments, which may be administered at various points in the curriculum. This article presents the results of a survey of U.S. schools of pharmacy regarding the use of cumulative assessments within their curriculum. A 20-question survey tool was emailed to 125 schools of pharmacy. A total of 105 out of 125 schools participated (response rate 84%). Of these, 52 schools currently have a cumulative assessment program; 18 have one cumulative exam prior to advanced pharmacy practice experiences (APPEs); 19 have a cumulative exam every didactic year; and seven have accumulative exams every semester, except during APPEs (*n* = 44). Increased faculty workload emerged as the top challenge faced by schools that have implemented a cumulative assessment program. Eighteen schools indicated that no outcomes are measured to determine the utility of the cumulative assessment. From these results, it appears that almost half of participating U.S. schools have implemented a cumulative assessment plan. However, it is apparent that more research needs to be done to determine which outcomes are expected to improve with the implementation of such an assessment plan.

## 1. Introduction

The Accreditation Council of Pharmacy Education (ACPE) has taken a strong stance on assessment within the pharmacy curriculum [1]. Current accreditation guidelines (Guideline 15.1) state that “In general, the college or school’s evaluation of student learning should: incorporate periodic, psychometrically sound, comprehensive, knowledge-based, and performance-based formative and summative assessments, including nationally standardized assessments (in addition to graduates’ performance on licensure examinations) that allow comparisons and benchmarks with all accredited and college or school-determined peer institutions.” The recent 2016 draft guidelines have expanded this edict by including language that assessment should evaluate student readiness to: (1) enter advanced pharmacy practice experiences (APPE); (2) provide direct patient care; and (3) contribute as a member of an interprofessional team [2]. This draft also makes reference to the use of the Pharmacy Curriculum Outcomes Assessment (PCOA), an optional, standardized, multiple choice exam developed by the National Association of the Boards of Pharmacy to measure student knowledge in different content areas. These draft ACPE guidelines clearly indicate that assessment of student achievement and readiness is an integral part of ensuring the quality of our pharmacy graduates. In making reference to PCOA, these guidelines also direct attention to the fact that a cumulative assessment tool is needed to assess student performance and to determine and document readiness for entrance into the APPE curriculum.

When considering assessment, there are several techniques that can assess student readiness and achievement of learning. These may include: “Written tests, oral examinations, student reflections, instructor evaluations, and performance in simulated settings.” [3]. However, most of these assessments are done within individual courses or course blocks. On the other hand, cumulative assessments or progression examinations are generally defined as assessments administered across the curriculum with the goal of measuring student acquisition and retention of knowledge that is based on a defined set of curricular outcomes [4,5,6,7,8,9,10,11,12,13,14]. Cumulative assessments are not linked to any one course, but instead measure the achievement of global curricular outcomes. These cumulative assessments may be used to: (1) identify students who may benefit from remediation exercises; (2) determine minimal competency to allow progression through the curriculum; (3) help ascertain retention of previous coursework; and (4) evaluate the overall curriculum by identifying curricular deficiencies. Data obtained from these assessments may also be used for benchmarking across various institutions. However, utilization of data for benchmarking may be difficult due to inherent differences in curricular sequencing across institutions [14].

Although extensive data are limited, some schools have instituted cumulative assessments at various points in their curriculum [4,5,6,7,8,9,10,11,12,13]. At the University of Houston, Sansgiry *et al.* published an article outlining their case-based written Milemarker exam, which is formative for the first two years and then summative in the third year, with the goal of ensuring student readiness for APPEs [4,5]. To ascertain student accountability, students are informed that failing the last part of Milemarker exam could result in delayed progression to APPEs [5]. Another paper written by Alston *et al.* described a cumulative assessment plan comprised of multiple choice questions designed to measure the acquisition of skills [6]. As part of this assessment plan, faculty have in place a rigorous process for assessing the reliability and validity of the cumulative exam, stressing that this is an important step in developing and implementing a cumulative assessment strategy. A more comprehensive plan was described by Me´sza´ros at Touro University-California, where a knowledge-based and objective-structured clinical exam (OSCE) is administered on an annual basis. Exam validity is ascertained by comparing exam results to APPE preceptor evaluations [7]. At the University of Kansas, Ragan and colleagues described a longitudinal OSCE administered annually to determine student competence for entrance into APPEs [9]. While most of these studies describe assessments that have been developed in-house, Scott and colleagues described the use of PCOA at Palm Beach Atlantic University [10]. The paper contended that the PCOA has great utility in identifying student and curricular deficiency, especially against national benchmarks. However, Scott also mentioned current limitations in using PCOA as a benchmarking tool. Scott argued that not only is participation across pharmacy schools limited, but student accountability varies across schools, making direct program comparison difficult and perhaps inaccurate.

Cumulative assessment utility has also been recommended in the literature by expert opinion and position papers, which further add support to their use in higher education [14,15]. Plaza published a paper recommending that schools use multiple types of cumulative assessments. When strategically placed after the establishment of knowledge and skill domains, these cumulative assessments afford institutions the opportunity to systematically measure students’ overall competence [14]. Plaza further argued that knowledge-based assessments may overly emphasize rote memorization of material; therefore, Plaza recommended the use of both knowledge and skill evaluations. Plaza opined that OSCEs would generate a clearer and more robust picture of individual student competence. When taking a composite of the literature reports and expert opinions, one common theme prevails: that cumulative assessments are opportunities to gain insight only when the assessment plans are founded on sound reliability and validity measures [6,7,14,15].

The last comprehensive evaluation and documentation of cumulative assessment utility in U.S. schools and colleges of pharmacy occurred in 1998 [16]. Data from that survey revealed that 19.6% (nine out of 77) of pharmacy programs administered cumulative assessments [16]. These results lack program representation considering that the number of pharmacy schools and colleges has nearly doubled since the study was conducted. Consequently, as evidence and literature bolsters the implementation of cumulative assessment in higher education, it is important to continue to collect data and document the progress of U.S. schools in implementing cumulative assessments within the pharmacy curriculum. To address recent dynamic changes within pharmacy education, we sought to survey all U.S. schools and colleges of pharmacy in order to gain insight about their experiences towards cumulative assessments. These data will provide valuable information to schools looking to develop or strengthen their existing assessment programs.

## 2. Experimental Section

A 20-question survey instrument consisting of 3 distinct evaluation sections was developed. Of the three sections, questions in Section 1 targeted school demographics and cumulative assessment utilization. Section 2 contained survey questions, which would aid in determining barriers to instituting cumulative assessments and the challenges faced by those schools that currently incorporate these assessments in their curriculum. Upon completing Section 1 and Section 2, only schools and colleges that have a cumulative assessment plan were asked to complete Section 3. Section 3 of the survey contained items designed to address assessment format, assessed topics, personnel responsible for plan development, validation methods utilized and, finally, outcome measurement.

The respondent pool for the survey instrument was identified through the American Association of Colleges of Pharmacy (AACP) faculty database, which included 125 partially- and fully-accredited U.S. colleges and schools of pharmacy. Inclusion criteria were individuals listed as chairs of the pharmacy practice department and/or individuals responsible for assessment. Schools that were identified as having pre-candidate status by ACPE were excluded from this study. The survey instrument was electronically administered through SurveyMonkey^®^ in April 2014 [17]. All data collected were input and collated in Microsoft Excel^®^ and analyzed using descriptive statistics. Participant consent was obtained at the beginning of the online survey. This study is approved by the University of the Pacific Institutional Review Board (Protocol No. 13-48).

## 3. Results and Discussion

### 3.1. Results

A total of 105 out of 125 schools participated in the study, a response rate of 84% (survey available as an Appendix). Respondent demographics are summarized in Table 1. Of the respondents, 53 (50.5%) reported not having or utilizing cumulative assessments; however, 13 of these (12.4%) reported that they are in the process of developing a cumulative assessment program. Of the 40 (38.1%) respondent programs without a cumulative assessment program, the most common reasons cited for “not” instituting such an assessment included “lack of resources” and that “knowledge assessed in individual courses is adequate” (Table 2).

**Table 1:** Demographics of respondent schools.

Specification		Have a Cumulative Assessment Plan?		
		Yes (*n* = 52)	No (*n* = 40)	In Development (*n* = 13)
Private School	Student Enrollment 100 or less	16 (30.7%)	8 (20%)	5 (38%)
	Student Enrollment >100	11 (21%)	10 (25%)	4 (30.7%)
Public School	Student Enrollment 100 or less	14 (27%)	11 (27.5%)	2 (15%)
	Student Enrollment >100	11 (21%)	11 (27.5%)	2 (15%)

**Table 2:** Reasons cited for “not” having a cumulative assessment plan.

Reason (Respondents Could Select More Than One)	*n* = 40
Lack of resources (faculty time, financial constraints, *etc.*)	23 (57.5%)
Knowledge is already assessed in individual courses	20 (50%)
Unconvinced it would add value to the current education program	17 (42.5%)
Unsure of what to do with the results	9 (22.5%)
Lack of evidence in the literature supporting its use	8 (20%)
Faculty have never considered it	5 (12.5%)

Of the fifty two (49.5%) schools that indicated having a cumulative assessment program within their curriculum, 19 (36.5%) have had cumulative assessments in place for more than five years. The most common reasons cited for implementing cumulative assessments included; “to ensure minimal competency in core educational outcomes” (77%), “help students identify deficiencies” (75%) and “ensure student accountability for developing a cumulative knowledge and skill set” (60%) (Table 3).

**Table 3:** Purpose of a cumulative assessment plan within the curriculum.

Purpose		*n* = 52
Student specific	Help students self-identify educational and learning deficiencies	39 (75%)
	Provide feedback to students affording them developmental opportunities	36 (69%)
	Ensure student accountability for developing a cumulative knowledge and skill set	31 (60%)
Program specific	Ensure minimal competency in meeting core educational outcomes	40 (77%)
	Identify curricular gaps and or deficiencies	32 (61.5%)
	Ensure a level of competence that is expected before student progresses in the program	30 (57.7%)

With regard to development of the cumulative assessment program, 31 schools involve all faculty members responsible for the delivery of content and material being tested, while 10 have a specific and dedicated cumulative assessment committee. For the remaining 12 schools, it is the assessment committee that is responsible for developing the cumulative assessment program. An overwhelming majority of schools (38 of 52) have an in-house assessment, whereas the remaining 14 have purchased a commercial question bank. As a composite, schools indicated that their assessments generally test knowledge/skills related to a variety of topic areas (Table 4).

**Table 4:** Areas assessed in the cumulative assessment plan.

Topic (Respondents Could Select More Than One)	*n* = 52
Therapeutics	43
Patient assessment	41
Pharmacy calculations	41
Medication counseling	41
Drug information and literature search	41
Kinetics	38
Pharmacology	37
Physiology	37
Medicinal chemistry	33
Law	32

As far as the timing and placement of the cumulative assessment plan, 18 schools only have one exam prior to APPEs; 19 schools have an exam every didactic year; and seven schools have a cumulative assessment every semester, excluding APPEs (*n* = 44, eight schools skipped this question). The format also varied, with 15 schools having both a written and oral exam, 29 having only a written exam and six having only an oral exam (*n* = 50, two schools skipped this question). Of the schools with an oral exam or oral exam portions, 14 use OSCEs to assess student performance; 14 assess patient interview/counselling skills; eight use simulations; and nine use an oral question and answer session (some schools use multiple formats). For those with a written exam or written exam component, 37 schools have a multiple choice exam question format; 18 utilize a case-based exam; while nine have a short answer/essay type exam, again with some schools using multiple formats.

With regard to the summative *vs.* formative nature of the assessments, 18 schools have both a low and high-stakes component; 19 have only a low-stakes exam with no consequence; and 10 schools have only high-stakes summative assessments in their curriculum (*n* = 47, five schools skipped this question). Of those with a high-stakes component, 11 schools indicated that students are given opportunities for remediation, but progression is halted if a student fails despite remediation efforts; four indicated that they offered unlimited remediation opportunities; and one indicated that failing the assessment results in a halt in progression without opportunity for remediation (*n* = 16, 12 skipped this question).

When asked about challenges to instituting a cumulative assessment plan, the majority cited “increased faculty workload” and “lack of evidence that these assessments actually improve long-term retention of knowledge” (Table 5). When asked about exam validation, 28 reported having no formal validation measures or standards in place. Of those claiming validation, seven validate by comparing performance on cumulative assessments with cumulative GPA, seven with national board exam pass rates, and 10 schools use validated package exams, such as the PCOA. When asked about outcome measurement since implementing the assessment, 18 schools indicated that no specific outcomes are measured. Of those who have measured outcomes, the most common responses were “improvements in board exam pass rates” (*n* = 5) and “performance on APPEs” (*n* = 5).

**Table 5:** Challenges faced by schools having a cumulative assessment plan within their curriculum.

Challenge (Respondents Could Select More Than One)	*n* = 52
Increased faculty workload	30 (57.6%)
Lack of evidence that progress exams actually improve long-term retention of knowledge	25 (48%)
Lack of exam validation	25 (48%)
Difficulty figuring out what to do with deficient students	24 (46%)
Inadequate remediation strategies	23 (44%)
Lack of student buy-in	15 (28.8%)
Lack of faculty buy-in	13 (25%)
No challenges faced	4 (7.7%)

### 3.2. Discussion

This study provides insight into the utilization of cumulative assessments in U.S. schools and colleges of pharmacy. Our results indicate that almost 50% of the surveyed schools have cumulative assessment in place, and an additional 12% are planning implementation. These numbers are substantially higher than the 19.6% reported in 2000 and may be reflective of the growing emphasis placed on learning as it relates to the achievement of curricular outcomes [16]. Nevertheless, it should be noted that the majority of schools with cumulative assessment plans have a low-stakes emphasis (no impact to progression). While low-stakes examinations avoid student stress and need for remediation, Szilagyi reported that the pass rate of the first Milemarker exam increased to 68.6% to 85.7% from 7% to 23.9% when the school added rewards and punishments to their formative assessment plan [11]. Therefore, when taken into consideration, low student effort and motivation can negatively impact performance, decreasing the validity of exam results [18]. To then find the right balance, it may be that a combination of both formative and summative assessments should be utilized as a means of assessing both student learning and curricular effectiveness [14].

In addition, based on one expert’s opinion, cumulative assessments should include both knowledge and skill assessments, so as to truly evaluate a student’s overall competence [14]. Despite these recommendations, very few schools (15) have implemented a plan that includes both of these components.

As the current 2007 ACPE standards do not provide guidance on the specific format for cumulative assessments, our results indicate that the schools have developed a variety of assessments administered at different time points in the curriculum. The advantage of this approach is that it gives schools the ability to develop assessments tailored towards their own curriculum, resource availability and knowledge domain acquisition timing. On the other hand, having each school devise individual cumulative assessments results in increased faculty work load and inability to benchmark and compare results across different programs. From the data collected, 10 schools indicated that their institutions have adopted PCOA. Published experience from other schools of pharmacy indicates that PCOA scores highly correlate with student GPA and may be a better measure of student knowledge than student perceptions of their own knowledge [10,12]. While the PCOA has the potential to provide meaningful information, there are also several current limitations associated with its use [10].

Although the majority of schools with a cumulative assessment plan reported several challenges associated with implementation, faculty workload was chosen as the leading challenge. Faculty engagement is needed for the plan to be successful, but the burden on faculty should be carefully balanced to avoid burn-out. Certainly, faculty involvement is substantial, as it involves plan development, implementation and maintenance, as well as ongoing effort and attention to question writing, grading/evaluation and remediation.

In addition to the assessment itself, another challenge faced by the schools is how to find an effective way to remediate students who “fail” the cumulative assessment or who show significant deficiencies in core content areas. The current health sciences literature indicates that early detection, development of an individualized remediation plan and assessment of the effectiveness of remediation can all be important pieces of the overall remediation strategy [19]. Additionally, the cumulative assessment plan itself can and should be part of the early detection system to identify and correct student deficiencies. In answering the “how to best remediate” question, our research was unable to identify any literature on the assessment of remediation strategies specifically related to cumulative assessments. Generally, the policies on academic progression vary widely among different schools, and there is little literature on the remediation strategies in pharmacy education [19].

With regard to impact on long-term retention, studies in the medical literature indicate that repeated testing results in repeated retrieval of memories and increases long-term retention [21,22,23]. Interestingly, almost half of the programs with cumulative assessment plans questioned both its validity and utility in improving long-term retention among students. As noted earlier, only a few schools have published validity (and reliability) findings of their cumulative assessments, and we were unable to identify studies that specifically evaluated long-term retention pre- and post-implementation of a cumulative assessment plan within pharmacy education.

Finally, student buy-in must be factored in as it can have a major impact on performance. As noted in the literature, students’ passing scores increase considerably in formative exams if a high-stakes incentive or punishment is in place [5,11]. In the article described by Sansgiry, the most productive incentive was carryover points towards a high-stakes exam [5]. One limitation of our study is that we did not specifically survey schools regarding incentive mechanisms currently in place. Surveying schools regarding incentive mechanisms would have provided valuable information for other programs looking to develop or improve their cumulative assessment plans.

## 4. Conclusions

Data gathered from this study show that almost half of U.S. schools and colleges of pharmacy have implemented cumulative assessment plans. However, these plans vary greatly in format and student accountability. Additionally, based on feedback, these assessment plans are fraught with challenges, including a lack of exam validity, a lack of student accountability and a lack of evidence demonstrating improved long-term retention. Unfortunately, the majority of schools do not attest that their use of cumulative assessments is tied to improvement in measured learning outcomes. Further study is needed to wholly justify the ongoing use of energy and resources that cumulative assessments require. Further study is also needed to determine the optimal format and sequencing of cumulative assessments so that minimal competency and student readiness for pharmacy practice is truly measured.

## Appendix

Survey Questions

1. What is the name of your program?
2. Please provide the most accurate description of your school/college.
  - Private University system
  - Public University
  - Private standalone
3. How many students do you enroll per class year?
4. Does your school/institution have or utilize comprehensive learning assessment(s)?
  - Yes
  - No
  - In development
5. Please click on the reason(s) your program does not use a comprehensive learning assessment. Check all that apply.
  - Lack of evidence in the literature supporting its use
  - Knowledge is already assessed in individual courses
  - Faculty have never considered it
  - Unsure of what to do with the results
  - Lack of resources (faculty time, financial constraints, etc)
  - Unconvinced it would add value to the current education program
  - Other
6. How long have you utilized a comprehensive learning assessment at your institution?
  - 1-2 years
  - 3-5 yeas
  - More than 5 years
7. What is the purpose of the comprehensive learning assessment? check all that apply
  - Ensure minimal competency in meeting core educational outcomes
  - Ensure student accountability for developing a cumulative knowledge and skill set
  - Help students self-identify education and learning deficiencies
  - Ensure a level of competence that is expected before student progresses in the program
  - Identify curricular gaps and or deficiencies
  - Provide feedback to students affording them developmental opportunities
  - Other
8. How many assessments do you have during the duration of your curriculum?
  - 1 per academic year
  - 1 per academic year except during APPEs
  - 1 per semester
  - 1 per semester except during APPEs
  - 1 exam only, prior progression to APPEs
9. Please describe the exam delivery style
  - Oral exam
  - Written exam
  - Both oral and written exam
10. If applicable, please describe the oral exam format. Check all that apply
  - Oral questions/answer session
  - Patient interview/counselling
  - Objective Structured Clinical Exam (OSCE)
  - Simulations
  - Not applicable
11. If applicable, please describe the written exam format. Check all that apply.
  - Short answer/essay
  - Multiple choice
  - Case based/Problem based solution (Progress note/SOAP note case, Drug information)
12. Who develops the comprehensive learning assessment every year?
  - All faculty involved in teaching that material
  - Comprehensive learning assessment faculty committee
  - Preceptors/practitioners
  - Administration
  - Assessment committee
  - Other
13. Which of the following best describes the goal of the comprehensive learning assessment?
  - High stakes summative assessment
  - Low stakes formative assessment
  - Both high and low stakes assessment
14. Which of the following best describes the consequence to those students who do not pass the exam? Check all that apply
  - Remediation one time then stop progression
  - Unlimited remediation
  - Stop progression
  - No consequence
15. Which of the following topics do you assess in the comprehensive learning assessment?
  - Therapeutics/clinical pharmacology
  - Kinetics
  - Pharmacology
  - Physiology/pathophysiology
  - Medicinal chemistry
  - Pharmacy calculations
  - Medication counselling
  - Drug information and literature research
  - Patient assessment
  - Law and regulation
16. What challenges have you faced with incorporating a comprehensive learning assessment into your curriculum? Check all that apply.
  - Increased faculty workload
  - Difficulty figuring out what to do with deficient students
  - Lack of exam validation
  - Lack of student buy-in
  - Lack of faculty buy-in
  - Inadequate remediation strategies
  - Lack of evidence that progress exams actually improve long term retention
  - No challenges faced
  - Other
17. Do you have a formal process to ensure validity of the assessment
  - Yes
  - No
18. If yes, what method do you use to validate the assessment?
  - Correlation with cumulative GPA
  - Correlation with board exam pass rate
  - Correlation with time to graduation
  - We use a question bank with validated questions
19. Where do you acquire questions for the exam? Check all that apply
  - Faculty provide questions
  - Use a pre-bought question bank
  - They are prior exam that have been recycled
20. Have you measured any outcomes since you instituted the comprehensive learning assessment?
  - Yes
  - No
  - If yes, please describe.
